# Environmental Pollution by Benzene and PM_10_ and Clinical Manifestations of Systemic Sclerosis: A Correlation Study

**DOI:** 10.3390/ijerph14111297

**Published:** 2017-10-26

**Authors:** Alice Borghini, Andrea Poscia, Silvia Bosello, Adele Anna Teleman, Mario Bocci, Lanfranco Iodice, Gianfranco Ferraccioli, Daniele Ignazio La Milìa, Umberto Moscato

**Affiliations:** 1 Institute of Public Health-Hygiene Section, Università Cattolica del Sacro Cuore, 00168 Roma, Italy; alice.borghini01@icatt.it (A.B.); andrea.poscia@unicatt.it (A.P.); ateleman@hotmail.com (A.A.T.); daniele.lamilia@gmail.com (D.I.L.M.); 2 Department of Rheumatology, Fondazione Policlinico Gemelli, 00168 Rome, Italy; silvia.bosello@libero.it (S.B.); mariobocci@gmail.com (M.B.); gff1990@gmail.com (G.F.); 3 Vice Medical Director, San Raffaele Cassino Hospital, 03043 Cassino (FR), Italy; lanfranco.iodice@sanraffaele.it

**Keywords:** systemic sclerosis, benzene, particulate matter (PM_10_), environmental exposure

## Abstract

Atmospheric air pollution has been associated with a range of adverse health effects. The environment plays a causative role in the development of Systemic Sclerosis (SSc). The aim of the present study is to explore the association between particulate (PM_10_) and benzene (B) exposure in Italian patients with systemic sclerosis and their clinical characteristics of the disease. A correlation study was conducted by enrolling 88 patients who suffer from SSc at the Fondazione Policlinico “A. Gemelli” in Rome (Italy) in the period from January 2013 to January 2014. The average mean concentrations of B (in 11 monitoring sites) and PM_10_ (in 14 sites) were calculated using data from the Regional Environmental Protection Agency’s monitoring stations located throughout the Lazio region (Italy) and then correlated with the clinical characteristics of the SSc patients. Of the study sample, 92.5% were female. The mean age was 55 ± 12.9 years old and the mean disease duration from the onset of Raynaud’s phenomenon was 13.0 ± 9.4 years. The Spearman’s correlation showed that concentrations of B correlate directly with the skin score (R = 0.3; *p* ≤ 0.05) and inversely with Diffusing Lung Carbon Monoxide (DLCO) results (R = −0.36; *p* = 0.04). This study suggests a possible role of B in the development of diffuse skin disease and in a worse progression of the lung manifestations of SSc.

## 1. Introduction

Atmospheric air pollution had been associated with a range of adverse health effects, particularly mortality and morbidity due to cardiovascular and respiratory diseases [1]. Moreover, numerous epidemiological studies have shown the higher incidence of these diseases in populations living in close proximity to highways, airports, and zones of high-density urban traffic [1,2]. Current evidence supports an interactive chain of events linking pulmonary and systemic pollution-induced oxidative stress, inflammatory events, and translocation of particle constituents with an associated risk of vascular dysfunction, atherosclerosis, altered cardiac autonomic function, as well as ischemic cardiovascular and obstructive pulmonary diseases [3]. In particular, fine particle pollution, such as particulate matter with a diameter ≤10 μm (PM_10_), is strongly associated with an increased risk of cardiovascular disease, deep vein thrombosis, and, as documented by the World Health Organization (WHO), a higher mortality rate, particularly in children and the elderly [4,5].

Benzene (B) is a well-known carcinogen of blood cells, yet both epidemiological and experimental studies suggest that B also interacts and determines mechanisms of toxicity for general health in humans, with particular reference towards diabetes and solid cancers [6].

Systemic Sclerosis (SSc) is a rare complex autoimmune-mediated disease associated with early inflammation, immune dysfunction, and vascular injury, followed by progressive fibrosis of the skin and internal organs, especially the lungs. Many independent lines of investigation suggest that the environment, acting on genetically susceptible individuals, can play a causative role in the development of such disease [7]. The role in the development of SSc of several occupational factors such as crystalline silica dust, white spirit, aromatic solvents, chlorinated solvents (i.e., trichloroethylene), ketones, and welding fumes has already been studied [8]. Studies show a higher prevalence of scleroderma in areas near British airports, suggesting a possible geographical clustering of the disease [9], the association between SSc and occupational exposure with a gender variability, and a risk of SSc which appears to be markedly associated with high cumulative exposure.

The aim of this study is to explore the association between the exposure to PM_10_ and B and the clinical and anamnestic characteristics of SSc in a group of patients referred to the Outpatient Clinic of the Rheumatology Department of the Fondazione Policlinico “A. Gemelli”.

## 2. Materials and Methods

A correlation study was conducted on patients with Systemic Sclerosis enrolled from January 2013 to January 2014 at the outpatient clinic of the Rheumatology Department of the Fondazione Policlinico “A. Gemelli” in Rome. Only patients who had been residents in the Lazio region of Italy for at least two years before the diagnosis of their condition were included in the study. In the study were enrolled 88 patients with a diagnosis of Systemic Sclerosis confirmed by a panel of Rheumatologists according to the criteria of the American College of Rheumatology [10].

The enrolled patients filled in a self-administered questionnaire, which included questions regarding demographics, residency, education, occupation, smoking, alcohol consumption, use of recreational drugs, and work and environmental exposures before and after the onset of the disease. The questionnaire was validated using the Delphi technique.

Moreover, the patients had to sign, at the time of enrollment, the informed consent form for the use of personal data. The study was conducted in accordance with the Declaration of Helsinki.

The patients were classified by two rheumatologists of the Fondazione Policlinico “A. Gemelli” Hospital according with the extension of cutaneous involvement into two categories based respectively on diffuse or limited skin disease (according to Leroy) [11] and on the presence or history of vascular ulcers. The patient skin involvement was assessed using the skin score technique (i.e., the sum of 10 body areas each scored by clinical palpation for tethering on a scale of 0–3). Lung involvement was assessed by measuring lung volume and the lung diffusion capacity for carbon monoxide (DLCO) through the DLCO test, performed according to the recommendations of the American Thoracic Society [12,13].

The B and PM_10_ concentration levels during the period from January 1999 to January 2014 were obtained from the Regional Environmental Protection Agency (ARPA Lazio) monitors present in different locations throughout the Lazio region. Only data derived from monitors located in the Province of Rome, which includes 11 monitoring sites for B and 14 sites for PM_10_, were considered. The annual mean concentration of B and PM_10_ was calculated for each monitor.

The locations of residence of each patient and the monitoring sites were geocoded using satellite maps. Subsequently, the distance between each patient’s residence and the monitoring sites was computed using ArcGIS software version 9 (ESRI, Redlands, CA, USA) and maps from the Geographic Information System of Lazio. After assigning each of the patients to the monitoring site which was nearest to their residence (for every year), the annual average concentrations of B and PM_10_ were calculated to asses the exposure.

The data on B and PM_10_ exposure in the two years before the onset of Raynaud’s phenomenon, available for some patients, were correlated with the demographic and clinical characteristics as well as the severity of disease.

Descriptive data were summarized using frequencies and percentages; continuous data were analyzed using means and standard deviations (Mean ± Standard Deviation). Differences in categorical or ordinal variables were evaluated through the chi-squared test or Fisher’s exact test, as appropriate. The differences in continuous variables were evaluated through Student’s *t*-test or the Mann-Whitney U Test, after assessing the normal distribution of the variables through the Shapiro-Wilk test. The association between the clinical severity of SSc patients and air pollutant annual concentration was investigated using Spearman’s rank correlation. A stepwise multiple linear regression was then performed using the following covariates: sex, age, education, occupation, smoking status, alcohol consumption, use of recreational drugs. A probability of *p* < 0.05 was considered statistically significant. All statistical tests were two-sided. Statistical analysis was performed using Stata IC 14 for Mac (Intercooled Stata 14.2 for MacIntosh, Stata Corporation, Lakeway, TX, USA, 2017).

The research described in our study was conducted as a routine activity in the network of outpatient service of Rheumatology of the Fondazione Policlinico “A. Gemelli” in Rome. Nevertheless data were collected anonymously by using the encrypted codes. Therefore, all the recruited persons were informed that participation was strictly voluntary, were assured that no information could lead to identification of any individual, and after reading the privacy policy statement those who were willing to participate in the study enclosed their firmed informed consents. Besides, our study was conducted in accordance with Helsinki Declaration.

## 3. Results

Nine people (among three different categories: two non-experts, three epidemiologists, and four rheumatologists) participated in a Delphi to validate the proposed questionnaire.

A score from 0 to 1 was assigned to each of the 19 questions in a given time in order to evaluate the comprehensiveness, pertinence, and objectivity of the questionnaire.

The overall agreement of the experts was approximately 94%. Due to the disagreement on 11 questions regarding their comprehensiveness, a few lexical modifications were introduced to improve the questionnaire. Given that substantial differences found in the evaluation of questions 3 and 19, these two questions were eliminated from the questionnaire. The Cohen’s K statistic to evaluate the inter-rater agreement among the observers was 0.8019 (*p* < 0.01). This reflected a substantial agreement, according to the existing literature [14].

Patient characteristics are shown in Table 1.

Eighty out of 88 selected patients filled in the questionnaire and were therefore included in the analysis. A very high percentage of the subjects (92.5%) were female. The mean age was 55 years (SD: 12.9) and the mean disease duration from the Raynaud phenomenon (RP) was 13.0 ± 9.4 years.

Fifty-nine patients (74%) were employed before the onset of SSc, while at the time of the questionnaire only 34 patients (43%) were still employed. Nineteen were retired at the time of our observation and 68% of these patients affirmed that retirement was related to disabilities deriving from SSc. Only four patients reported using paint and/or solvents in their free time. As shown in Table 1, a cumulative 79.49% of the subjects were living within 250 m of a high source of air pollution. Forty-six patients (57.5%) lived in a big city (Rome).

The concentrations of B and PM_10_ in the two years before the onset of RP were available for 33 patients. The mean distance between area of residency and the nearest monitor was 10.5 (±9.6) km for PM_10_ and 10.8 (±9.9) km for B.

**Figure 1:**
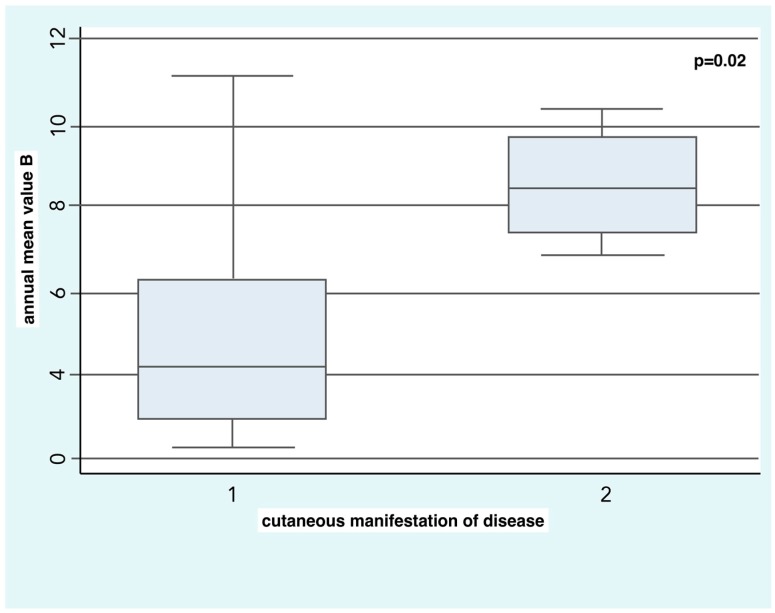
Box plot of mean annual Benzene (B) concentration in patients with diffuse skin disease and limited skin disease (*p* = 0.02).

SSc patients with diffuse skin disease were exposed in the two years before the onset of RP to higher concentrations of B (8.5 ± 1.5 µg/m^3^) compared to the patients with limited skin disease (4.9 ± 2.7 µg/m^3^) with a statistical significance of *p* = 0.02 (Figure 1).

**Figure 2:**
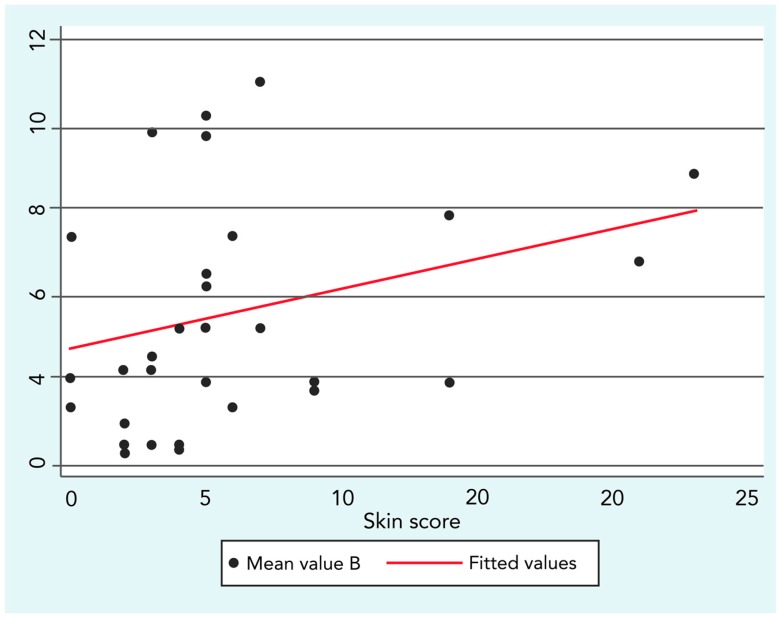
Spearman’s rank correlation between Benzene (B) and skin score (R = 0.3; *p* ≤ 0.05).

This finding was confirmed by a low but significant positive correlation between B exposure and skin score (R = 0.3; *p* ≤ 0.05), as shown in Figure 2.

Furthermore, SSc patients with active ulcers or history of digital ulcers were exposed in the two years before the onset of the RP to higher concentrations of B (6.4 ± 3.2 µg/m^3^) compared to the patients without ulcers or history of ulcers (4.9 ± 2.3 µg/m^3^). However, the difference was not statistically significant.

The analysis regarding SSc lung impairment and environmental exposure demonstrated a significant yet low negative correlation between B concentrations and DLCO values (R = −0.36; *p* ≤ 0.05).

**Figure 3:**
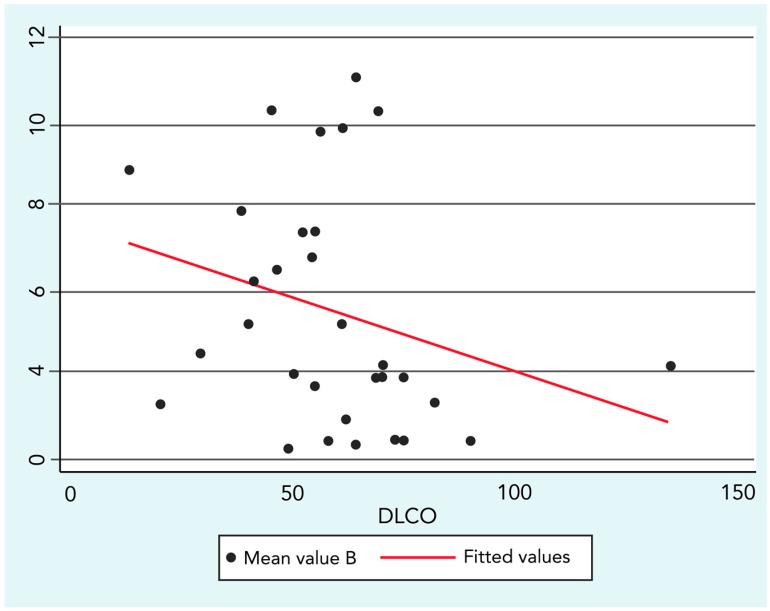
Spearman’s rank correlation between Benzene (B) concentration and Diffusing Lung Carbon Monoxide (DLCO) (R = −0.36; *p* ≤ 0.05).

Figure 3 graphically represents the correlation and its linear fit in a scatter-plot.

No correlations or significant differences were found in the different subgroups of patients as regards PM_10_. A stepwise multiple linear regression was then run using the covariates as stated in the methods section, but it did not show any statistical significance.

## 4. Discussion

Although there are critical issues in establishing an association between exposure and disease outcome in rare chronic diseases such as scleroderma [15], our study suggests that environmental exposure, in particular to B, could influence the severity of SSc. The association between this disease and exposure to environmental agents, such as toluene and B, is supported by several case reports and case-control studies [16]. This is the first study that compared environmental exposure to B in a SSc cohort according to clinical characteristics, and B exposure seems to be associated with a more aggressive and severe disease. In fact, patients with diffuse skin disease, which usually is associated with a more frequent internal organ involvement and worse outcome [17], were exposed in the two years before the onset of RP (the first sign of SSc) to higher B concentrations. This suggests that B could have a role not only in the manifestation of the disease, but also in the determination of its clinical characteristics. The correlations between B concentration and skin score and DLCO further support this hypothesis. In fact, we can speculate that a higher the environmental exposure determines a higher vascular inflammatory-fibrotic burden, influencing the extension of the skin disease and the lung damage measured through DLCO. A study analyzed scleroderma in patients exposed to trichloroethylene, B, toluene, xylene dieseline, and an amine component of epoxy resins [bis(4-amino-3-methylcyclohexyl)methane], suggesting that these compounds may cause SSc through the metabolism of the epoxy compound that subsequently binds certain proteins, creating autoantigens [18]. Vascular damage is considered the earliest pathogenic moment in SSc. B could represent an ulterior further damage mechanism which causes on endothelial dysfunction, as supported by the slightly higher B exposure in SSc patients with digital ulcers [19]. On the other hand, it is believed that B exerts its adverse effects through the metabolic activation of toxic metabolites. In fact, certain B metabolites are genotoxic and mutagenic [20]. B is classified as a Group 1 carcinogen to humans and animals by the International Agency for Research on Cancer (IARC), but its role in the development of cancer in scleroderma patients should be further investigated in other studies. Interestingly, patients with diffuse skin disease have a higher risk of cancer compared to other scleroderma patients and, as seen from our results, these patients are exposed to higher B concentrations [21].

Recently, an Italian group demonstrated that the exposure to diesel engine exhaust nanoparticulate induces the expression of inflammatory cytokines and fibroblast chemical mediators on SSc skin keratinocytes and fibroblasts [22]. Our data on air pollution exposure did not support a role of PM_10_, despite the fact that air pollution has been consistently linked to increased risk of cardiovascular disease and a higher prevalence of scleroderma in boroughs in close proximity to major British airports [1,2,9].

As far as limits are concerned, our study enrolled a small sample size with a high female/male ratio (12, 33:1); however, this reflects the epidemiology of the disease as shown in literature [23]. Such a limit probably affects the lack of statistical significance when performing a linear regression model. A larger sample would probably return a higher chance of finding any statistical differences, where they exist. Another limit of this work is the absence of healthy individuals so as to compare the exposure levels of our patients with the exposure levels of a control sample in order to better define this relationship. The investigation of the relationship between SSc severity and air pollution exposure is limited to Lazio, as the majority of the patients enrolled in this study were residents in Lazio. Furthermore, the presence of limited monitoring stations for particulate matter in some Italian regions, in particular regarding the PM_2.5_ monitoring stations, reduced the geographic diffusion of the present study [24,25].

Another limitation is represented by the apparently high dependence of the correlation by some outlier values. Unfortunately, removing these observations from the analysis causes a loss of statistical significance. On the other hand, data on the B and PM_10_ concentrations were obtained by an institutional agency, so we assume that all observations are validated with an international standard and every monitoring site reflects the real “state of the art” of the area surrounding that particular sensor. Given that we could not demonstrate a causative nature of the B and PM_10_ air pollution with the available data, we can still rely on this association in support of our findings.

In this study, the questionnaire was useful to collect and analyze data regarding the patients’ daily habits and the impact of the disease in their life. This tool could be improved in order to investigate other determinants related to the onset and progression of Systemic Sclerosis.

## 5. Conclusions

Our study shows that B concentration is correlated with skin involvement extension and DLCO levels in SSc patients, thus suggesting a possible role of air pollution exposure in determining the severity of the disease and of its lung involvement. This result is consistent with previous literature suggesting that particular matter could determine chronic lung disease [26].

In conclusion, this study suggests a possible role of B in the development of diffuse skin disease and in a worse progression of the lung manifestations of SSc. Additional evidence in a larger multicenter observational study is needed to confirm our results.

## Figures and Tables

**Table 1:** Patient characteristics.

Characteristics	Total Population (n = 80)
Age *mean* (SD)	55 (12.9)
Sex %	
Male	7.5
Female	92.5
Disease duration * *mean* (SD)	13.0 (9.4)
Smoke %	
Smokers	16
Ex smokers	25
Never smokers	59
Residency area **	
<20 m	42.31
20–100 m	26.92
100–250 m	10.26
>250 m	20.51

* mean disease duration from the Raynaud phenomenon in years; ** distance between residence area and a high-capacity road.
